# Modulation of NMDA receptor activity by CR4056, an imidazoline-2 receptor ligand with analgesic properties

**DOI:** 10.3389/fpain.2022.1003068

**Published:** 2022-09-22

**Authors:** Giulia Puja, Gabriele Losi, Lucio Rovati, Marco Lanza, Gianfranco Caselli, Rita Bardoni

**Affiliations:** ^1^ Department of Life Sciences, University of Modena and Reggio Emilia, Modena, Italy; ^2^ Department of Physics, Institute of Nanoscience -CNR (CNR-NANO S3), Modena, Italy; ^3^ Rottapharm Biotech, Monza, Italy; ^4^ Department of Biomedical, Metabolic and Neural Sciences, University of Modena and Reggio Emilia, Modena, Italy

**Keywords:** NMDA receptor, pain, electrophysiology, neuronal cultures, spinal cord, imidazoline-2 receptor ligands

## Abstract

CR4056 is an imidazoline-2 receptor ligand having potent analgesic activity and synergistic effect with opioids. Very recently it has been found that CR4056 can revert the cognitive impairment in animal models of Alzheimer's disease (AD). Since several lines of evidence highlight the importance of NMDAR modulators in nociceptive signaling and in AD progression, we considered as important to investigate the effects of CR4056 on NMDAR activity. In primary culture of cortical neurons, application of NMDA and glycine elicits a current that is decreased in a dose-dependent fashion by CR4056 (IC_50_ 5.3 ± 0.1 µM). CR4056 antagonism is reversible, not competitive and voltage-independent and it is not blocked by pertussis toxin. CR4056 interacts with the co-agonist glycine site in a competitive way, indeed high glycine concentrations diminish its effect. Fibroblasts expressing different recombinant NMDA receptors are differently modulated by CR4056: the potency and the efficacy of the compound are higher in GluN1- GluN2B than in GluN1-GluN2A containing receptors. In lamina II neurons of spinal cord slices, single stimulation of afferent fibers evokes an NMDA-mediated current that is inhibited by 10 µM CR4056. Repetitive stimulation of the dorsal root at high frequency and high intensity produces a firing activity that is significatively depressed by CR4056. Taken together, our results broad the understanding of the molecular mechanisms of CR4056 analgesic activity, involving the modulation of NMDAR activity. Therefore, we propose that the analgesic action of CR4056 and the neuroprotective effects in AD models may be mediated also by NMDAR inhibition.

## Introduction

L-Glutamate is the major excitatory neurotransmitter in the mammalian CNS and its signal transduction is mediated by different membrane receptors such as metabotropic and ionotropic (iGluR) receptors. IGluR are composed of four major subtypes, α-amino-3-hydroxy-5-methyl-4-isoxazolepropionic acid (AMPA), Kainic Acid (KA), N-methyl-D-aspartate (NMDA) and delta receptors, having important roles in several physiological and pathological situations (1). NMDA receptors (NMDARs) are widely distributed throughout the central and peripheral nervous system and participates in the sensory processing and transmission of pain, as well as in synaptic plasticity and central sensitization of neuropathic and inflammatory pain (2). NMDARs are heterotetramers derived from the assembly of three subunits, GluN1, GluN2 and GluN3, where GluN1 and GluN2 are essential for the formation of functional channels (3–5). The GluN1 subunit is localized throughout the dorsal horn of the spinal cord, including the laminae that play an important role in pain transmission (6). All GluN2 subunits (A, B, C and D) are expressed in superficial dorsal horn: recordings of synaptic responses from lamina I-II neurons have shown that GluN2 A/B are the main subunits expressed at synapses (7, 8). While GluN2A mediates synaptic transmission in acute pain, GluN2B seems to play a major role in conditions of neuronal disinhibition, that occurs in some chronic pain conditions (9). Consistently, GluN2B antagonists decrease nerve injury-induced mechanical allodynia (10) related to a reduced inhibition in dorsal horn (11). CR4056 (2-phenyl-6-(1H-imidazol-1yl)quinazoline) is an imidazoline-2 receptor (I2R) ligand with a potent and broad analgesic activity in several animal models of inflammatory, chronic, and neuropathic pain (12–14). The molecular mechanisms of CR4056 action leading to analgesia had been investigated *in vivo* and *in vitro* in rat dorsal root ganglion cells: CR4056 inhibits PKCε translocation (15) a cellular event involved in pain peripheral sensitization and targeted also by other analgesics. Lately a selective synergism in the analgesic effect between CR4056 and morphine was demonstrated and their combination showed an improved safety and abuse liability profile over morphine alone (16). Furthermore, in phase II clinical trial CR4056 showed analgesic activity in humans (17) and more recently, it has been demonstrated that CR4056 has beneficial effects on neuroinflammation, and on spatial memory in a mouse model of Alzheimer's disease (AD) (18).

Because compounds able to modulate NMDA receptor activity have important analgesic and neuroprotective properties the goal of this work was to investigate CR4056 effect on NMDAR- mediated currents.

## Materials and methods

### Animals

The Italian Ministry of Health approved all the experiments conducted on postnatal rats of either sex following the Guide for the Care and Use of Laboratory Animals and the EU and Italian regulations on animal welfare.

### Neuronal primary cultures

Primary cultures of cortical (CX) and cerebellar (CB) neurons were prepared from newborn (CX) or seven days old (CB) Sprague-Dawley rats Briefly, cells from cortex and cerebellum were dispersed with trypsin (0.24 mg/ml; Sigma Aldrich, Milan, Italy) and plated at a density of 0.8 × 10^6^ cells/ml on 35 mm Falcon dishes coated with poly-L-lysine (10 µg/ml, Sigma Aldrich). Cells were plated in basal Eagle's Medium (BME; Celbio, Milan, Italy), supplemented with 10% fetal bovine serum (Celbio), 2 mM glutamine, 25 mM KCl and 100 µg/ml gentamycin (Sigma Aldrich) and maintained at 37°C in 5% CO_2_. After 24 h *in vitro*, the medium was replaced with 1:1 mixture of BME and Neurobasal medium (Celbio, Milan) containing 2% B27 supplement, 1% antibiotic, and 0.25% glutamine (Invitrogen). At 1days *in vitro* (DIV5), cytosine arabinofuranoside (Ara-C) was added at final concentration of 1 µM. Thereafter, half of the medium was replaced twice a week with Neurobasal medium containing 2% B27 supplement, 1% antibiotic, and 0.25% glutamine.

### JM4C cells culture

Mouse connective tissue fibroblasts JM4C cells stably transfected with NR1A/NR2A or NR1A/NR2B human NMDA inducible receptors were kindly donated by Dr. Paul Whiting, Merk Sharp / Dohme (UK). They were grown in Dulbecco's Modified Basal Eagle's medium (DMEM) with Na+ pyruvate, 4,500 mg glucose and glutamine. For growing, DMEM was added with geneticin 1 mg/ml (Sigma) and foetal bovine serum 10% (Celbio). One day before experiment, fibroblasts confluent dishes were split with trypsin-EDTA 0.05% (Euroclone) and plated at the density of 0.5 × 10^6^ cells/ml in 2 ml dishes containing an inducing DMEM with ketamine (final concentration 0.015%, Gellini International, Italy) and 25 nM dexamethasone (Sigma Aldrich) to induce NMDA receptors expression.

### Spinal cord slices

Sprague–Dawley rats (male and female at postnatal days P15-P23) were anaesthetized with isoflurane and decapitated. The spinal cord and vertebrae were rapidly removed and placed in ice-cold dissecting Krebs' solution (composition in mM: 90 NaCl, 50 sucrose, 2.5 KCl, 25 NaHCO_3_, 1 NaH_2_PO_4_·H_2_O, 25 glucose, 6 MgCl_2_, 1.5 CaCl_2_, 1 kynurenic acid), bubbled with carboxygen (95% O_2_, 5% CO_2_). The lumbar part of the spinal cord was isolated, embedded in low melting point agarose (3% w/v, Thermo Fisher Scientific, Waltham, United States), and transverse slices (500 µm thick) were obtained using a vibrating microtome (WPI, Sarasota, United States). Slices were incubated in oxygenated incubation Krebs' solution (composition in mM: 125 NaCl, 2.5 KCl, 25 NaHCO_3_, 1 NaH_2_PO_4_·H_2_O, 25 glucose, 6 MgCl_2_, 1.5 CaCl_2_) at 32°C for 30 min and then used for recording.

### Electrophysiological recordings

Recordings were performed in cortical and cerebellar neurons after 7–9 days in culture or in spinal cord slices from postnatal rats. All recordings were performed at room temperature, under voltage-clamp in the whole-cell configuration of the patch-clamp technique (19). Electrodes for recordings from neurons in culture were pulled from borosilicate glass (Hidelberg, FRG) on a vertical puller (PB-7, Narishige) and had a resistance of 5–7 Ohm when filled with KCl internal solution. Currents were amplified with an Axopatch 1D amplifier (Axon Instruments, Foster City. CA), filtered at 5 kHz, digitized at 10 kHz.

Neurons in spinal cord slices were visualized using an Axioskop microscope (Zeiss, Oberkochen, Germany), fitted with Nomarski optics and connected to a CCD camera (Dage-MTI, Michigan City, United States). Patch-clamp recordings were performed by using thick-walled borosilicate pipettes (3–5 MOhm resistance). Data were recorded and acquired using a MultiClamp 700A amplifier and the pClamp 10 software (Molecular Devices, Sunnyvale, United States). Sampling rate was 10 kHz, and data were filtered at 2–5 kHz. The dorsal root attached to each slice was stimulated using a suction electrode. Stimulus duration was 0.1 ms, stimulus intensity was 500 µA, able to activate both A and C primary afferent fibers (20). Extracellular field potentials were recorded by positioning a glass pipette (3–5 µm diameter, filled with Krebs' extracellular solution) on lamina II in spinal cord slices. Extracellular synaptic potentials were evoked by dorsal root stimulation, as described above, and amplified by using a Multiclamp 700A amplifier (Molecular Devices, Sunnyvale, United States).

### Solutions and drugs

The chamber used for recording from cultured neurons was continuously perfused at 2 ml/min with an extracellular medium composed of (mM): 145 NaCl, 5 KCl, 1 CaCl2, 5 Hepes, 5 Glucose, and 20 Sucrose, pH 7.4 with NaOH. Intracellular solution contains (mM): 140 KCl, 3 MgCl2, 5 EGTA, 5 Hepes, and 2 ATP-Na, pH 7.3 with KOH.

For the experiments on neurons in slices the following Krebs' extracellular solution was used (composition in mM): 125 NaCl, 2.5 KCl, 25 NaHCO_3_, 1 NaH_2_PO_4_·H_2_O, 25 glucose, 1 MgCl_2_, 2 CaCl_2_. A cesium-based internal solution was used during the voltage-clamp experiments (composition in mM: 120 caesium methanesulfonate, 10 sodium methanesulfonate, 10 EGTA, 1 CaCl_2_, 10 Hepes, 5 lidocaine *N*-ethyl bromide quaternary salt-Cl, 5 MgATP, pH adjusted to 7.2 with CsOH, osmolarity 290 mOsmol L^−1^), while a potassium-based solution was utilized for the experiments in current-clamp (composition in mM: 120 potassium methanesulfonate, 10 NaCl, 10 EGTA, 1 CaCl_2_, 10 Hepes, 5 MgATP, pH adjusted to 7.2 with KOH, osmolarity 300 mOsmol L^−1^).

All the components of Krebs' and intracellular solutions for slice recording and 2-BFI were obtained from Sigma-Aldrich (Merck Group, Darmstadt, Germany). NBQX, bicuculline methiodide, and strychnine hydrochloride were provided by Abcam (Cambridge, UK). CR4056 was provided by Rottapharm Biotech (Monza, Italy). The vehicle of CR4056 was tested in preliminary experiments and was not producing any effect on NMDA-evoked current.

All drugs were applied directly by gravity through a Y-tube perfusion system (21) in the experiments performed in culture while in slice experiments CR4056 and the receptor antagonists were bath perfused.

When CR4056 (10** **µM) was delivered inside the cell through the patch pipette the response to NMDA application was recorded at time 0 (just after breaking inside the cell) and after 1.5, 3, 4.5, 6 min. The NMDA-evoked current was recorded at these time intervals and the amplitude of the current was measured.

### Data analysis

The effect of CR 4056 in primary neuronal cultures was calculated as % variation of the NMDA-evoked current after application of the drug. A negative variation means a reduction of the control current. Off-line data analysis, curve fitting, and figure preparation were performed with Clampfit 9 or 10 (Molecular Devices, Sunnyvale, CA, United States), Origin 4.1 (Microcal, Northampton, MA, United States), and Microsoft Office (Microsoft). SigmaPlot 11 (SYSTAT, Palo Alto, CA, United States) and GraphPad Prism 9.3 (GraphPad Software, San Diego, CA, United States) were used for statistical analysis.

All data were expressed as the arithmetic mean ± standard error of the mean (SEM).

## Results

### CR4056 reduces NMDA-evoked currents in neuronal primary cultures

**Figure 1:**
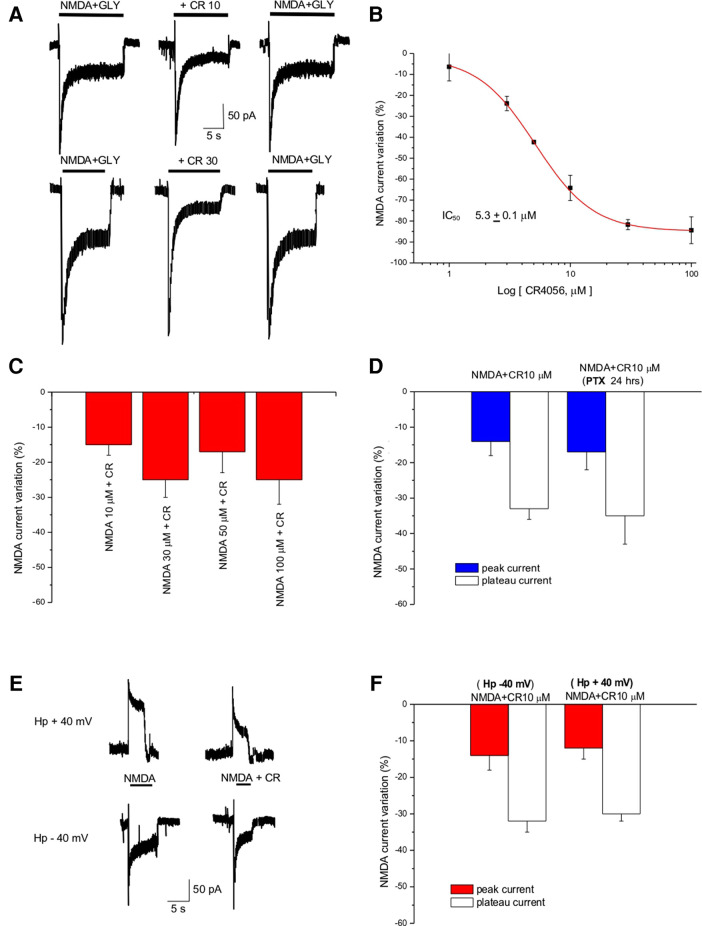
CR4056 decreases NMDA-evoked current in cortical cultures. (**A**) Representative traces showing the effect of CR4056 (10 µM and 30 µM) on the current evoked by NMDA (100 µM) + Glycine (Gly, 10 µM) application. (**B**) Dose response curve of the effect of CR4056 on NMDA plateau current. Each point is the mean ± SE of 5–7 experiments. (**C**) Histogram summarizing the effect of CR4056 (10 µM) on the current elicited by increasing concentrations of NMDA. (**D**) Effect of CR4056 on NMDA- peak and plateau current in control and after 24 h incubation with PTX (200 ng/ml, preincubated for 24 h) (**E**) Representative traces showing NMDA-evoked current before and after CR4056 (10 µM) application in a neuron held at −40 mV (lower trace) and at +40 mV (upper trace). (**F**) Histogram displaying the reduction of NMDA peak and plateau current after CR4056 application at −40 or +40 mV. Each histogram bar is the mean ± SE of 6 cells. In the experiments of panels (**C**–**E**) no significant differences were detected between groups (*p* > 0.05).

NMDA-evoked currents were recorded in primary culture of cortical neurons using the patch-clamp technique in the whole cell configuration. Fast application of NMDA (100 µM) and Glycine (10 µM) elicited an inward current that was decreased by CR4056 (Figure 1A); the effect was reversible and dose-dependent (Figure 1B). The IC_50_ derived from the dose-response curve is 5.3 ± 0.1 µM and the maximal reduction of NMDA current is 84.4 ± 6%. (Figure 1B).

CR4056 (10 µM) dialyzed inside the cell *via* the patch pipette (see Methods) did not change significantly (*p* > 0.05, *t*-test) the amplitude of NMDA currents (data not shown) suggesting that the substance does not bind to the NMDA receptor from the intracellular side.

To investigate the type of antagonism of CR4056, increasing concentrations of NMDA (from 10 to 100 µM) were applied together with CR4056 (10 µM). CR4056 reduced to the same extent the current evoked by increasing NMDA concentration suggesting that its antagonism is not competitive (Figure 1C).

To rule out the possibility that the effect we detect could be mediated by a Gi protein activation we incubated the neuronal cultures with pertussis toxin (PTX 200 ng/ml for 24 h). There were no statistically significant differences between the CR4056 reduction of NMDA currents measured in control and after PTX (Figure 1D).

To highlight a possible voltage dependence of CR4056 we analyzed the reduction of NMDA-evoked current at different holding potentials: CR4056 effect was not significatively different at positive and negative potentials (−31 ± 1% at +40 mV; −33 ± 4% at – 40 mV) (Figures 1E,F).

**Figure 2:**
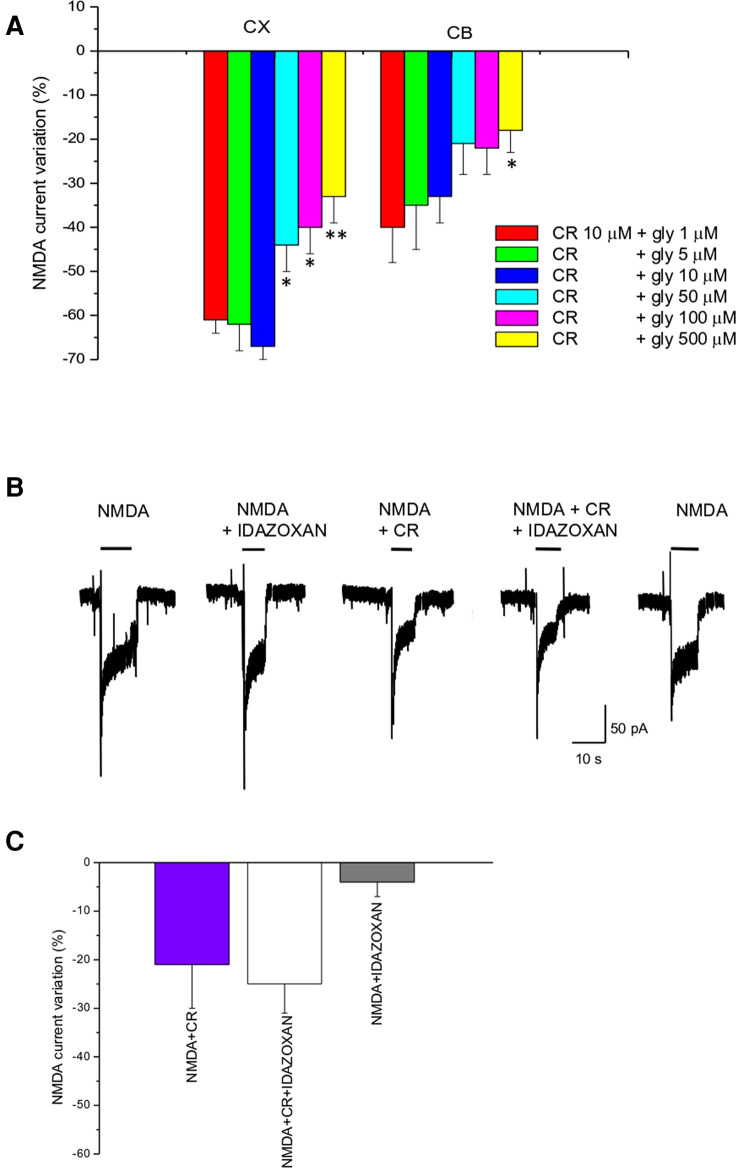
CR4056 effect is glycine dependent and is not affected by idazoxan. (**A**) Histogram depicting the effect of 10 µM CR4056 on NMDA-evoked currents (plateau) recorded in cortical (CX) and cerebellar (CB) neuronal cultures in the presence of increasing concentrations of glycine. Histogram bars are the mean ± SE (*n* = 5–11). There are statistically significant differences among groups (One-way ANOVA). Asterisks mean *p < 0.05; **p < 0.01, vs. 1 µM glycine (**B**) Electrophysiological recordings showing the effect of idazoxan (10 μM) on NMDA-evoked currents when is applied alone or together with 10 µM CR4056. Idazoxan did not affect NMDA-elicited current by itself (second trace) and did not block the effect of CR4056 when co-applied with the compound (forth trace). (**C**) Histogram showing the reduction of NMDA current after CR4056, idazoxan and the two compounds together. Histogram bars are the mean ± SE (*n* = 6). No significative differences were detected (*p* > 0.05, *t*-test).

Glycine is an important co-agonist of the NMDA receptor. To test the dependence of CR4056 modulation on glycine concentration we measured the reduction of NMDA current by CR4056 (10 µM) in the presence of 1, 5, 10, 50, 100, and 500 µM glycine. The effect of CR4056 was significatively reduced (one-way ANOVA Test) at high glycine concentrations both in cortical and in cerebellar neurons (Figure 2A).

*In vivo* experiments (12) showed that the analgesic effect of CR4056 was antagonized by administration of idazoxan, an imidazoline I2R ligand. To rule out the possibility that I2Rs could be in some way involved in the CR4056 effect at the level of NMDAR we co-applied idazoxan together with CR4056. As shown in Figure 2B, application of idazoxan (10 µM*)* does not change CR4056 effect.

Previous papers have shown that compounds able to selectively abolish the current mediated by NMDA receptors containing the NR2B subunit have good analgesic properties and no side effects (22, 23), for this reason we tested the effect of CR4056 on recombinant NMDA receptors.

### CR4056 modulation in fibroblasts expressing GluN1-GluN2A or GluN-GluN2B subunits

**Figure 3:**
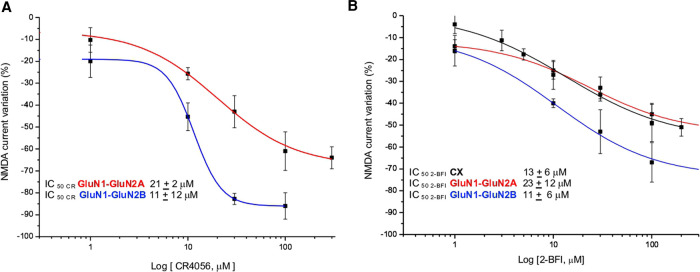
CR4056 modulation in fibroblasts expressing different subunits of NMDA receptor. (**A**) Dose response curves of the reduction of NMDA-evoked current by CR4056 on receptors containing GluNR1-GluNR2A (red line) or GluNR1-GluNR2B (blu line) (**B**) Dose response curves of 2-BFI effect on NMDA current in cortical neurons (CX, black line) and in fibroblasts expressing GluNR1-GluNR2A (red line) or GluNR1-GluNR2B (blue line) subunits. Each data point is the mean ± SE of 4–7 cells. In the inset the IC50 values of CR4056 and 2-BFI in the diverse cell cultures.

Increasing concentrations of CR4056 (0.1–100 µM) were applied to NMDA-evoked currents in fibroblasts expressing GluN1- GluN2A or GluN1- GluN2B subunits (Figure 3A). Both potency (IC50) and efficacy (Eff) of CR4056 are higher in fibroblasts bearing GluN1- GluN2B receptors compared to GluN1-GluN2A containing receptors (IC_50CR4056/**2A**_ = 21 ± 12 µM; IC_50CR4056/**2B**_ = 11 ± 2 µM; Eff_CR4056**/2A**_ = −47 ± 3%, Eff_CR4056/**2B**_ = −86 ± 6).

2-BFI, 2-(2-Benzofuranyl)-2-imidazoline hydrochloride, is a high affinity I_2_ R ligand reported, *in vitro,* to act as a fast, non-competitive and reversible inhibitor of NMDA receptors (24). For matter of comparison with CR4056 we studied the effect of increasing concentrations of 2-BFI in native and recombinant NMDA receptors. 2-BFI dose dependently reduces peak and plateau NMDA currents in cortical neurons with an IC_50_ of 8 ± 2 µM and an efficacy of 76 ± 2% (Figure 3B). The potency of 2-BFI in fibroblasts expressing GluN1- GluN2B and GluN1- GluN2A receptors is 11 ± 6 µM and 23 ± 12 µM, respectively. Similarly to CR4056, the maximal effect of 2-BFI is significantly greater when the GluN2B subunit is present (Eff_2−BFI/**2A**_** **= −39 ± 6%; Eff_2−BFI/**2B**_** **= −73 ± 7%; *p* > 0.05, *t*-test) (Figure 3B).

### Modulation of NMDA mediated synaptic responses by CR4056 in spinal cord slices

**Figure 4:**
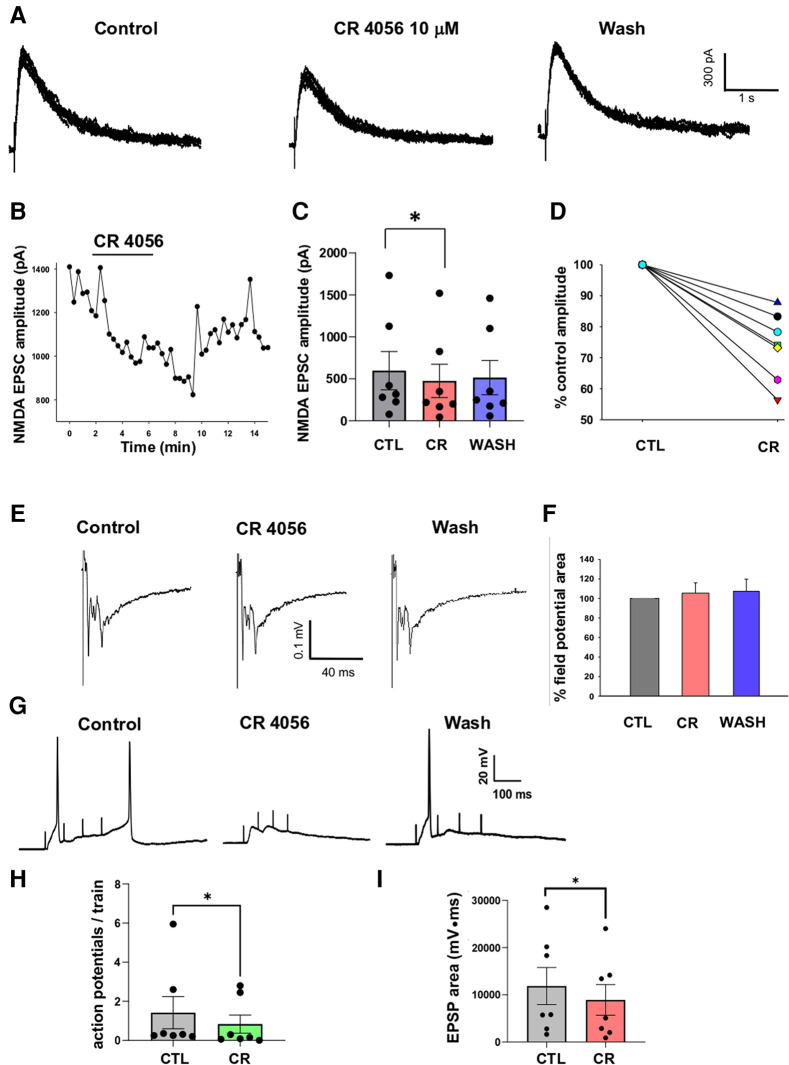
Application of CR 4056 depresses NMDA mediated synaptic responses evoked in rat dorsal horn lamina II. (**A**) Examples of NMDA EPSCs recorded from a lamina II neuron in rat spinal cord slices (7–8 consecutive traces are superimposed). NMDA currents were evoked by stimulating the dorsal root and were recorded in voltage-clamp at +40 mV, in the presence of NBQX (10 µM), Bicuculline (10 µM) and Strychnine (0.5 µM). 5 min application of CR 4056 (10 µM) caused the decrease of NMDA EPSC amplitude, that was partially reversible in some neurons. (**B**) Time course of the CR 4056 effect on the EPSCs shown in A. Bar indicates the duration of CR 4056 application. (**C**) CR 4056 significantly depressed NMDA EPSC amplitude in the sample of lamina II neurons tested compared to control (CTL) (One-way ANOVA repeated measures test, followed by Tukey test CTL vs. CR: *p* = 0.02; CTL vs. wash and CR vs. wash: *p* > 0.05; *n* = 7.). (**D**) Percentage changes of NMDA EPSC amplitude observed in six different neurons (represented by the different lines).”. (**E**) Examples of field potentials, extracellularly recorded from rat dorsal horn laminae I-II and evoked by root stimulation. (**F**) Histogram showing the percentage field potential area (mean +/− SE) in the presence of CR4056 (CR) and after washing (WASH), compared to control (CTL). Field potential area was not changed after CR 4056 (One-way ANOVA repeated measures test, *p* > 0.05, *n* = 6). (**G**) Recordings obtained from a lamina II neuron in current clamp, by stimulating the dorsal root with 4 consecutive pulses at 10 Hz. Application of CR 4056 decreased the summated EPSPs and the firing of action potentials (APs). (**H,I**) The average number of APs generated at each train (**H**) and the area of the summated EPSPs (**I**) were significantly reduced by CR4056 (**H**: Wilcoxon Signed Rank test, *p* = 0.01, *n* = 7; **I**: Paired *t*-test, *p* = 0.01, *n* = 7). See also Supplementary Materials for raw data and statistical analysis.

We next investigated the modulatory effect of CR4056 on NMDA-mediated postsynaptic excitatory currents (EPSCs) recorded from lamina II neurons in rat spinal cord slices. Glutamate release from primary afferent fibers (of both A and C type) was evoked by stimulating the dorsal root attached to the slice. To isolate NMDA EPSCs (recorded in voltage clamp at +40 mV), AMPA, GABA_A_ and glycine receptors were blocked by NBQX (10 µM), bicuculline (10 µM), and strychnine (0.5 µM), respectively. The electrophysiological recording of Figure 4A shows the effect of CR4056 (10 µM) on NMDA EPSCs: CR4056 significantly reduced the NMDA EPSC amplitude and a partial recovery was obtained in some neurons (Figure 4B). The average reduction in current amplitude was of 26.3 ± 4.2% from a sample of 7 neurons (Figures 4C,D).

To determine whether the effect of CR4056 on NMDA EPSCs was due to a decrease of glutamate release from primary afferents or to a modulation of NMDARs, we recorded extracellular field potentials evoked by dorsal root stimulation (Figure 4E). As shown in previous studies, field potentials recorded from spinal cord dorsal horn are mainly mediated by AMPARs (25, 26). Application of CR4056 did not affect field potential area in 6 tested slices (Figure 4F), suggesting a postsynaptic effect of the compound on NMDARs.

Finally, we tested whether CR4056 was able to depress lamina II neuron excitability in response to repetitive stimulation of the dorsal root. Recordings in current-clamp were obtained from 7 lamina II neurons at the neuron resting potential. The dorsal root attached to the slice was stimulated with 4 pulses 5–10 Hz, at the intensity of 500 µA. This stimulation protocol evoked summated EPSPs, mediated by both AMPARs and NMDARs (previous observation), that were able to generate action potentials (Figure 4G). Application of 10 µM CR4056 decreased both the area of the summated EPSPs (−29.7 ± 5.4%) and the mean number of action potentials generated at each train (−50.9 ± 12.6%). Comparison between the values obtained in control and CR4056 revealed that the compound produced a significant reduction of both parameters (Figures 4H, 4I).

## Discussion

NMDA receptors are key elements in pain transmission, indeed in pathological situations, such as neuropathic pain, an overactivation of these receptors was demonstrated (26).

CR4056 is an I2 R ligand with strong analgesic activity that was proved in different animal models of inflammatory, chronic and neuropathic pain (12–14).

Here we show that CR4056 have antagonistic activity at the level of NMDAR evoked currents.

In primary culture of cortical neurons CR4056 reduces NMDA-mediated currents with a potency in the low micromolar range. Its effect was reversible, not competitive and voltage independent. Application of the compound from inside or incubation with PTX failed to modify NMDA-evoked currents suggesting that its action was not mediated by an intracellular site nor dependent on G_i_-protein pathways (Figure 1).

The NMDA receptors, composed of GluN1/GluN2, have a stringent requirement of simultaneous binding of glycine or D-serine to GluN1 and glutamate to GluN2 (3). The glycine site is usually not saturated and changes in D-serine released from astrocytes or variation in glycine reuptake or release from nearby glycinergic terminals could influence NMDA receptor function (3). Indeed antagonists at the glycine site of the NMDAR have anti-hyperalgesic actions in experimental neuropathic pain (27).

Contrary to the *in vivo* studies (12), in our experimental model CR4056 effect was not blocked by idazoxan (Figure 2C) suggesting that the I2R is not involved in the CR 4056 modulation of the NMDAR activity.

The NMDA receptor is composed of different subunits (GluN1-3) (1, 3) and phosphorylation of the GluN1 subunit at protein kinase C (PKC) and protein kinase A (PKA) - dependent sites plays a key role in enhancement of NMDA receptor activity in the spinal cord related to pain transmission and to the processes of central sensitization and pathogenesis of neuropathic pain (28). It has been shown that GluR2B subunit selective compounds have good analgesic properties with little side effects (10, 21, 22). Indeed, intrathecal administration of GluN2B antagonists decreases certain forms of chronic pain including PGE2 or NMDA induced allodynia (29), capsaicin or carrageenan-induced hyperalgesia (23) and nerve injury-induced mechanical allodynia (10). The finding that CR4056 has higher potency and efficacy on receptors containing GluN2B subunit than on those assembled from GluN2A subunit (Figure 3) is suggestive of a good pharmacological profile.

2-BFI is a ligand of I2R reported to act as a fast, non-competitive and reversible inhibitor of NMDARs *in vitro* (24). More recent work demonstrated that 2-BFI attenuates hypersensitivity and spinal neuroinflammation in a rat model of neuropathic pain (30) and that the antinociceptive effects involve intracellular Ca^2+^ elevation and/or downstream Ca^2+^/calmodulin signaling (31).

In our experiments, performed on cortical neurons, 2-BFI showed an IC_50_ of 13 ± 6 µM and a maximal reduction of NMDA-evoked current of 48 ± 5%, while in recombinant NMDARs containing GluN2A and GluN2B subunit the IC_50_ was 23 ± 12 µM and 11 ± 6 µM, respectively. The efficacy was lower when the GluN2A subunit was present in the receptor assembly (Eff_2−BFI/**2A**_** **= −39 ± 6%; Eff_2−BFI/**2B**_** **= −73 ± 7%).

On the basis of the present results, we can hypothesize that the decrease in NMDA current after CR4056 application derives from a direct action on specific sites of the NMDAR. As previously mentioned, the current reduction measured after CR4056 application is probably a result of an increase in NMDAR desensitization. Several different mechanisms can underly NMDAR desensitization including, among others, the glycine-dependent desensitization and calcium-dependent inactivation (3). The degree of fast NMDA-receptor desensitization is inversely related to glycine concentration, since NMDA responses desensitize very little at high glycine concentrations (>10 µM; 27). CR4056 could increase NMDAR desensitization by displacing glycine from its site, suggesting that the glycine unbinding rate determines this form of desensitization.

Ca^2+^ entry into the cytosol during NMDAR activation could also trigger Ca^2+^-dependent desensitization of NMDARs, and CR4056, similarly to what shown for other drugs (32), could promote this process. Furthermore, it has been reported that intracellular calcium signaling plays an important role in the antinociceptive activity of 2-BFI (31), whose mechanism of action is similar to that of CR4056. In neuropathic pain an increase in NMDAR phosphorylation produces an intensification in NMDAR activity leading to hyperalgesia (28). Vellani et al. demonstrated that CR4056 reduces PKCε translocation on activated sensory neurons both *in vitro* and *in vivo*. The inhibition of PKCε translocation in cultured DRG was very fast (30 s), blocked by PTX and insensitive to idazoxan. The *in vitro* action of CR4056 was confirmed also *in vivo* and was consistent with the analgesic effect measured after CR4056 administration (15). In the same paradigm 2-BFI was ineffective in blocking PKCε translocation but was able to potentiate CR4056 effect when applied at sub-optimal concentrations suggesting a synergism in the action of the two compounds (15).

Collectively these data prompt us to speculate that the analgesic effect of CR4056 is achieved thanks to different mechanisms; among them a reduction of NMDAR mediated current could derived by a decrease in NMDAR phoshorilation due to inhibition of PKCε translocation (15) and from an action at the level of the receptor, presumably at the glycine site.

Data obtained from spinal cord slices confirm the inhibiting effect of CR4056 on NMDARs observed in cultured neurons. We show that application of the compound reversibly decreases the amplitude of NMDA-mediated EPSCs evoked by dorsal root stimulation. The EPSCs were recorded from neurons located in lamina II, an area receiving a prevalence of nociceptive inputs. Furthermore, the dorsal root attached to the slice was stimulated at high intensity (500 µA), able to recruit both Aδ and C nociceptive afferents. Our results, showing a significant depression of glutamatergic synaptic responses in superficial dorsal horn in the presence of CR4056, are consistent with the analgesic effects of the compound reported in previous studies (12–14).

The effect of CR4056 on spinal cord slices was likely due to a direct inhibition of postsynaptic NMDARs, in agreement with the data obtained from cultured neurons. Indeed, extracellular field potentials evoked by dorsal root stimulation were not affected by CR5046. Since these signals represent the sum of glutamatergic responses originated in lamina II neurons at their resting potential, they are almost completely mediated by AMPA receptors. Thus, the lack of effect on field potentials indicates that, in our experimental conditions, the compound does not modulate glutamate release from primary afferents nor alters AMPAR function expressed on dorsal horn neurons.

NMDARs have been involved in several mechanisms of spinal pain sensitization, being important regulators of superficial dorsal horn synaptic plasticity and excitability (reviewed in 33–35). Spinal NMDARs critically contribute to phenomena of amplification and potentiation of the nociceptive input, such as wind-up and LTP, which generate the state of neuronal hyperexcitability typical of several forms of persistent pain (36, 37). We have tested the effect of CR4056 on summated synaptic responses evoked by repetitive stimulation of the dorsal root. The frequency of stimulation applied in our experiments (5–10 Hz) is believed to resemble the frequency afferent barrage occurring in C fibers under pathological states (37). Summated EPSPs and superimposed action potentials were significantly depressed by CR4056, acting on NMDARs. This result suggests that CR4056 can be effective in reducing NMDA-mediated hyperexcitability of dorsal horn neurons, that develops in chronic pain conditions.

Our study has been carried out on naïve rats, showing that CR4056 is effective in inhibiting pain transmission at the spinal level. Further studies would be required to test the analgesic effect of CR4056 in pathological conditions, in animal models of both inflammatory and neuropathic pain. In particular, studies performed *in vivo* will be helpful to clarify the NMDA dependency of CR4056 action.

Since NMDARs take part in spinal microglia activation in peripheral inflammatory pain and hyperalgesia in rats (38) and changes in microglia profiles by CR4056 were detected, resulting in a suppression of pro-inflammatory activated microglia (18), it is possible that CR4056-mediated inhibition of microglial NMDARs contributes to its pharmacological effects. Indeed, it has been demonstrated that CR4056 has beneficial effects on neuroinflammation, BBB functionality, and spatial memory in a mouse model of AD presenting heavy amyloid load, neuronal and memory loss (18). Seen the pivotal role played by the NMDA dysfunction in AD (39) and the use of memantine, a non-competitive NMDAR antagonist, in moderate to severe AD, it is suggestive to believe that the mechanisms of action involving NMDARs highlighted in the present study, probably contributing to the pain relieving activity of CR405, may also partially explain its protective action in AD models.

In conclusion our results provide novel mechanism of action for CR4056 at the level of NMDAR that could contribute to its analgesic and neuroprotective properties *in vivo*.

## Data Availability

The raw data supporting the conclusions of this article will be made available by the authors, without undue reservation.
