# Supplementary material for: Modulation of NMDA receptor activity by CR4056, an imidazoline-2 receptor ligand with analgesic properties
Source: Front Pain Res (Lausanne). 2022 Sep 22;3:1003068. doi: 10.3389/fpain.2022.1003068 (PMC9632859; doi:10.3389/fpain.2022.1003068)
Supplement: Supplementary file 1 [file Datasheet1.pdf]

Table 1: CR4056 effect on evoked NMDA EPSCs (Figure 4C) + analysis in Sigmaplot 11

	EPSC amplitude in CTL (pA)	EPSC amplitude in CR (pA)	EPSC amplitude in WASH (pA)
Cell 1	419.1	349.3	352.0
Cell 2	78.5	44.0	57.3
Cell 3	227.3	168.2	175.3
Cell 4	1128.2	826.1	1100.0
Cell 5	1732.0	1521.0	1460.2
Cell 6	318.0	200.4	211.0
Cell 7	280.3	219.1	249.1

One Way Repeated Measures Analysis of Variance

Normality Test: Passed (P = 0.258)

Equal Variance Test: Passed (P = 0.977)

Treatment Name	N	Missing	Mean	Std Dev	SEM
EPSC ctl	7	0	597.629	603.559	228.124
EPSC CR	7	0	475.443	525.034	198.444
EPSC wash	7	0	514.986	540.093	204.136

Source of Variation	DF	SS	MS	F	P
Between Subjects	6	5531745.919	921957.653		
Between Treatments	2	54419.932	27209.966	5.618	0.019
Residual	12	58123.161	4843.597		
Total	20	5644289.012			

The differences in the mean values among the treatment groups are greater than would be expected by chance; there is a statistically significant difference (P = 0.019). To isolate the group or groups that differ from the others use a multiple comparison procedure.

Power of performed test with alpha = 0.050: 0.665

All Pairwise Multiple Comparison Procedures (Tukey Test):

Comparisons for factor:

Comparison	Diff of Means	p	q	P	P<0.050
Ctl vs CR	122.186	3	4.645	0.017	Yes
Ctl vs wash	82.643	3	3.142	0.108	No
Wash vs CR	39.543	3	1.503	0.554	No

Table 2: CR4056 effect on action potential firing and EPSP area (Figures 4 H and I) + analysis in Sigmaplot 11

	N Action potentials/train in CTL	N Action potentials/train in CR	EPSP area in CTL (mV*ms)	EPSP area in CR (mV*ms)
Cell 1	0.25	0.15	2818.5	2049.0
Cell 2	0.31	0.06	5821.5	2887.5
Cell 3	0.25	0.00	20159.0	14156.0
Cell 4	5.95	2.80	28492.3	24012.2
Cell 5	0.36	0.30	5746.4	5089.4
Cell 6	0.20	0.08	1646.8	878.7
Cell 7	2.60	2.45	18324.7	13400.2

Figure 4H: Paired t-test:

Normality Test: Failed (P < 0.050)

Wilcoxon Signed Rank Test

Group	N	Missing	Median	25%	75%
N AP CTL	7	0	0.310	0.250	2.040
N AP CR	7	0	0.150	0.0650	1.913

W= -28.000 T+ = 0.000 T-= -28.000
Z-Statistic (based on positive ranks) = -2.371
P(est.)= 0.022 P(exact)= 0.016

The change that occurred with the treatment is greater than would be expected by chance; there is a statistically significant difference (P = 0.016).

Figure 4 I: Paired t-test:

Normality Test: Passed (P = 0.159)

**Treatment Name**	**N**	**Missing**	**Mean**	**Std Dev**	**SEM**
EPSP area ctl	7	0	11858.457	10385.863	3925.487
EPSP area CR	7	0	8924.714	8545.954	3230.067
Difference	7	0	2933.743	2248.414	849.821

t = 3.452 with 6 degrees of freedom. (P = 0.014)

95 percent confidence interval for difference of means: 854.307 to 5013.179

The change that occurred with the treatment is greater than would be expected by chance; there is a statistically significant change (P = 0.014)

Power of performed test with alpha = 0.050: 0.793
